# Supplementary material for: Activation of neuronal adenosine A1 receptors causes hypothermia through central and peripheral mechanisms
Source: PLoS One. 2020 Dec 16;15(12):e0243986. doi: 10.1371/journal.pone.0243986 (PMC7743955; doi:10.1371/journal.pone.0243986)
Supplement: Attachment — Submitted filename: PONE response to reviews v4.docx [file pone.0243986.s002.docx]

Edward E Schmidt
Academic Editor
PLOS ONE

November 20, 2020

**RE:** PONE-D-20-31332
**Title:** "Activation of neuronal adenosine A1 receptors causes hypothermia through central and peripheral mechanisms"

Dear Dr Schmidt and reviewer,

We thank you and the reviewer for their insightful and thoughtful comments, which have improved the manuscript. All of the comments are addressed below (reviewer’s comments in black and our responses in blue) and shown as 'track changes' in a supplemental file.

Regards,

Marc

Editor:

In particular, I would like you to improve the introduction for clarity and to address the concerns about the missing positive control for the experiment shown in Figure 6.
All points are addressed, as detailed below.

Reviewer #1: Major points: This article explores the relationship between an adenosine receptor (A1AR), regions of the brain and hypothermia. Overall, the article would be much stronger and would benefit from more in-depth explanation on what the target of the study is. The last line of the introduction (line 75) indicates that the study will be looking at where A1AR agonist will act to cause hypothermia, and this is assuming the authors mean where in the brain, but there is not enough introduction about what is known currently about A1AR/hypothermia/brain localization in the intro. Where is A1AR localized in the brain? What regions are believed to control hypothermia and which have not been explored? As the authors noted, other studies have also “suggested a role in hypothermia for A1AR in the preoptic area and other brain regions”, so what is unique about this study?
We have completely re-written the Introduction to address all of these points, and think it is much improved. Thanks.

Intro - It should be clarified that the Adora1 locust encodes the protein of interest, A1AR.
Fixed
Figure 1 – Significance of the data should be noted with p values
Added in Fig 1 legend
Line 239 – “Since A1AR are widely” change are to “is”
Fixed, here and several other places in the manuscript.
Figure 3, a new abbreviation (A1KO) is used, but this is not discussed in the rest of the paper or defined. Authors should add definition or use Adora-/- or Adorafl as in the rest of the paper.
As suggested, we changed A1KO in the figure to match *Adora1-/-* in the legend.
Line 229 – consider rephrasing “A1AR agonist-induced hypothermia is also caused by acting on brain neurons.” It is unclear in the currently written form
Changed to “This demonstrates that A_1_AR agonists can cause hypothermia by acting directly on brain neurons.”
Figure 5 – This figure would benefit from labeling of different brain regions, as in the other figures, especially the noted cortex, hippocampus, and thalamus
We have added labels and inserts to the figure and defined them in the figure legend.
Figure 6 – A positive control demonstrating that the AAV8-hSyn-DIO-hM4Di-mCherry does cause hypothermia in other areas of the brain would be beneficial. Alternatively, some evidence that the hM4Di inhibitor is actually being expressed in addition to the mCherry would be beneficial. It is impossible to know if the hM4Di didn’t cause hypothermia in the POA or if the hM4Di segment is inactive. Also, some explanation of the uneven distribution of mCherry in Figure 6A would be beneficial. Is the VMPO special for some reason that it has a lot of AAV8-hSyn-DIO-hM4Di-mCherry present?
We added to the Methods “This batch of AAV8-hSyn-DIO-hM4Di-mCherry was functional in other experiments in our lab that were not part of this project.” We also added “The hM4Di-mCherry is a fusion protein, so if mCherry is expressed, then hM4Di is also expressed.” (<http://www.ncbi.nlm.nih.gov/pubmed/21364278>). This rules out a viral tropism problem, such as if the virus did not infect cells expressing Adora1-Cre. The uneven distribution of the mCherry is due to the inherent variability of viral injections. This likely explains the variability seen with CNO in Fig 7C.

Figure 7 – Comparison of Fig7B and 7E would be easier with a clear title or label of the variable that is changed (testing activation in the POA vs DMH).
As suggested, we now label both Fig 7B and 7E, and also updated the Figure legend.
Lines 276 and 279 – the wording “at least unilateral” should be changed to just “unilateral”, there’s nothing more than unilateral.
changed to “unilateral or bilateral”
Line 280 – The authors should clarify that while they induced hyperthermia, it is not necessarily given that the same region and neuron subpopulation would control hypothermia.
We completely agree with the reviewer, and think the wording reflects this uncertainty “Thus, the DMH is a potential candidate region for neurons driving A1AR agonist-induced hypothermia”.
Figure 7B – Fix “CNO” in legend.
Fixed
