# Supplementary material for: Activation of neuronal adenosine A1 receptors causes hypothermia through central and peripheral mechanisms
Source: PLoS One. 2020 Dec 16;15(12):e0243986. doi: 10.1371/journal.pone.0243986 (PMC7743955; doi:10.1371/journal.pone.0243986)
Supplement: Attachment — Submitted filename: response to editor 2020.11.30.docx [file pone.0243986.s003.docx]

From the Editor:

“I see that you have not included a statement in the text about public availability of the mouse models used in this study. I presume that the null and floxed alleles of Adora1 that you used can be obtained by contacting Drs. Schnermann and Greene, respectively, via the information given on the manuscripts you have cited for these models. Can you please revise the manuscript, however, to instruct readers how they can obtain the ADAR1Cre mouse line that you developed in this study?”

Indeed, the null and floxed alleles of Adora1 can be obtained by contacting Drs. Schnermann and Greene, respectively.

We have added a sentence (line 104) “The Adora1-Cre mouse is available from the corresponding author.” With acceptance of the manuscript, we will deposit the mouse in a repository.
